# Function and evolution of microRNAs in eusocial Hymenoptera

**DOI:** 10.3389/fgene.2015.00193

**Published:** 2015-05-27

**Authors:** Eirik Søvik, Guy Bloch, Yehuda Ben-Shahar

**Affiliations:** ^1^Department of Biology, Washington University in St. LouisSt. Louis, MO, USA; ^2^Department of Ecology, Evolution, and Behavior, The Alexander Silberman Institute of Life Sciences, The Hebrew University of JerusalemJerusalem, Israel

**Keywords:** miRNA, Aculeata, Hymenoptera, eusociality, non-coding RNAs

## Abstract

The emergence of eusociality (“true sociality”) in several insect lineages represents one of the most successful evolutionary adaptations in the animal kingdom in terms of species richness and global biomass. In contrast to solitary insects, eusocial insects evolved a set of unique behavioral and physiological traits such as reproductive division of labor and cooperative brood care, which likely played a major role in their ecological success. The molecular mechanisms that support the social regulation of behavior in eusocial insects, and their evolution, are mostly unknown. The recent whole-genome sequencing of several eusocial insect species set the stage for deciphering the molecular and genetic bases of eusociality, and the possible evolutionary modifications that led to it. Studies of mRNA expression patterns in the brains of diverse eusocial insect species have indicated that specific social behavioral states of individual workers and queens are often associated with particular tissue-specific transcriptional profiles. Here, we discuss recent findings that highlight the role of non-coding microRNAs (miRNAs) in modulating traits associated with reproductive and behavioral divisions of labor in eusocial insects. We provide bioinformatic and phylogenetic data, which suggest that some Hymenoptera-specific miRNA may have contributed to the evolution of traits important for the evolution of eusociality in this group.

## Introduction

Most insect species are solitary, and behavioral interactions with conspecifics are primarily restricted to reproductive behaviors such as male–female courtship and male–male competition. This is in sharp contrast to social insects, where groups of genetically related individuals often live together in a colonial lifestyle. The size and stability of these colonies vary from a few individuals sharing a nest for a short period of time, to large perennial colonies composed of thousands of individuals (Hölldobler and Wilson, 2009). The most advanced form of animal social organization is termed “eusociality” (Crespi and Yanega, 1995), marked by the presence of sterile workers that often forgo own reproduction in order to support the reproduction of other colony members. Although eusociality is relatively rare in most taxonomic animal lineages, eusocial species have been immensely successful. Current projections estimate eusocial insects to represent the largest proportion of the global animal biomass (Hölldobler and Wilson, 2009). Although the reasons for this remarkable success are not well-understood, it is commonly assumed that the social lifestyle of these animals must have played a major role in their current ecological dominance (Wilson, 1990). For example, it is thought that specialization in task performance (division of labor) amongst eusocial workers enables colonies to maximize the exploitation of their environment. In contrast, solitary insects have to multitask independent activities, including foraging and brood rearing (Wilson, 1985).

The recent sequencing of genomes from diverse social and solitary Hymenoptera clades offers a unique opportunity for identifying genome-level molecular events that may have supported the emergence of specific traits associated with the evolution of eusociality (“eusocial traits”). The ability to compare whole-genome sequences, gene expression patterns, and other molecular properties of species with diverse forms of social lifestyles, has generated novel mechanistic and evolutionary insights into these complex behaviors. This approach has been used most successfully in studies of the division of labors in worker tasks (Smith et al., 2008) and reproduction (Schwander et al., 2010), both of which are hallmarks of eusociality (Wilson, 1985). To date, the efforts to decipher the evolution of eusocial traits, and the mechanisms that support them, have focused on protein-coding genes (Keller and Ross, 1998; Page and Amdam, 2007; Fischman et al., 2011; Woodard et al., 2011). In contrast, how non-coding regulatory RNAs may have played a role in the evolution of eusociality is understudied. Here, we examine the emerging role of an important class of small, non-coding RNAs, which are collectively referred to as “microRNAs” (miRNAs), in regulating social behaviors. We discuss their possible role in regulating eusocial traits in social Hymenoptera at the developmental, physiological, and evolutionary time scales.

## miRNAs: History and Background

During the early days of the molecular biology revolution, the majority of research on gene regulation was limited to transcriptional mechanisms of protein coding genes as originally defined by the “Central dogma of molecular biology” (Crick, 1970). However, the discovery of the regulatory function of non-coding RNAs indicated that the early views on gene regulation and their associated phenotypic outcomes, were oversimplified and required major revisions to the dogma. We now know that in addition to transcriptional regulation (Lee and Young, 2000; Yan et al., 2015), gene functions are also regulated by factors such as post-transcriptional RNA editing (Gott and Emeson, 2000), mRNA splicing (Breitbart et al., 1987), RNA degradation (Bushati and Cohen, 2007), and diverse post-translational protein modifications (Braakman and Bulleid, 2011). More recently, regulatory non-coding RNAs have also emerged as important factors that regulate phenotypic variation via diverse molecular mechanisms (Qureshi and Mehler, 2012; Bonasio and Shiekhattar, 2014).

**FIGURE 1:**
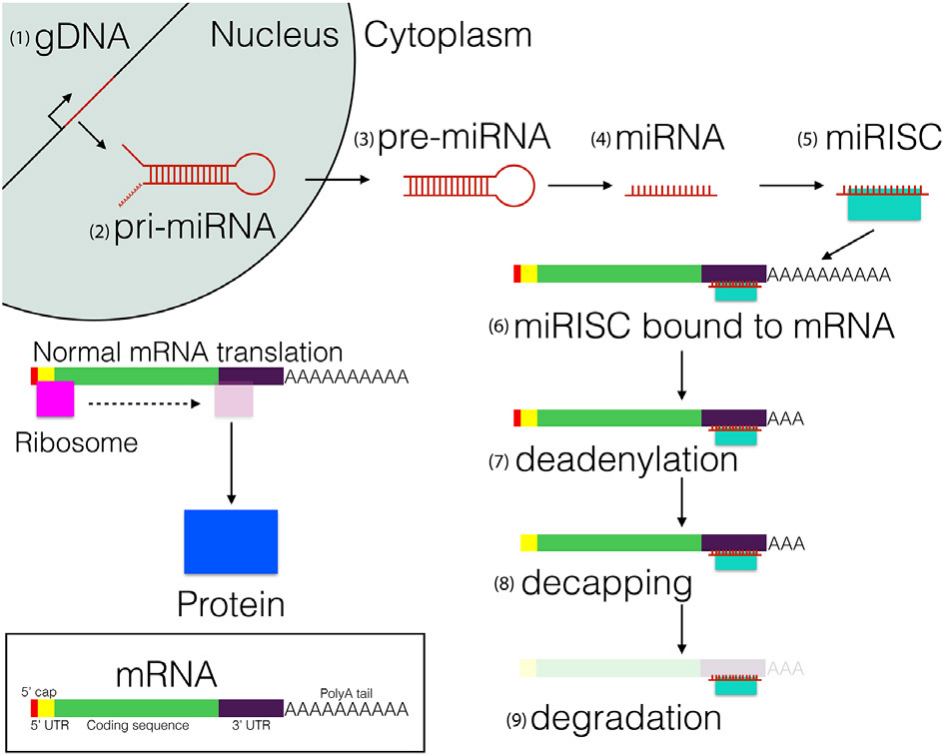
**MicroRNA biosynthesis, processing, and function**. (1) miRNA are transcribed as 80–100 nucleotide (nt) hairpin loops. (2) The initial transcript, referred to as the primary-miRNA (pri-miRNA), (3) is cleaved into precursor miRNA (pre-miRNA) and exported to the cytoplasm. Subsequently, (4) the pre-miRNA is cleaved into a single mature miRNA strand, (5) which binds to the RNA-Induced Silencing Complex (miRISC, shown in turquoise). (6) The miRISC binds to the 3′UTR of mRNAs, which leads to the inhibition of protein translation. Eventually the mature mRNA becomes (7) deadenylated and (8) decapped, which leads to transcript degradation by RNases.

miRNAs are short (18–24 nucleotides) non-coding RNAs, which in animals seem to act primarily by repressing protein translation via interaction with the 3′UTR of mRNAs (**Figure 1**). miRNAs were first discovered in the nematode *Caenorhabditis elegans*, where the miRNA *cel-lin-4* was shown to be necessary for the temporal timing of key developmental events (Lee et al., 1993). Because of their short length and the nature of their molecular interaction with mRNA targets, it has been hypothesized that a single miRNA can potentially regulate the function of multiple protein-coding genes (Bartel, 2009), and thus act as a pleiotropic genetic factor (Bartel, 2004). It is estimated that between one and two thirds of mRNAs encoded by animal genomes are regulated by miRNAs (Berezikov, 2011). As a result, it is likely that miRNAs play some roles in the regulation of most biological processes in animal cells (Bushati and Cohen, 2007).

## miRNAs in Development and Function of Nervous Systems

Various miRNAs have been implicated in neuronal development (Alvarez-Garcia and Miska, 2005; Wienholds and Plasterk, 2005). There is evidence that miRNAs play important roles in fine tuning the temporal and spatial regulation of protein translation during development (Aboobaker et al., 2005; Wienholds et al., 2005). For example, miRNAs have been shown to affect canonical signaling pathways that are important for nervous system development, such as the MAPK and Notch signaling pathways (Lai et al., 2005; Chiba, 2006; Louvi and Artavanis-Tsakonas, 2006; Paroo et al., 2009; Zhu et al., 2010). It has been hypothesized that in these essential developmental pathways, miRNAs reduce the impact of stochastic variability in mRNA transcript levels on actual protein levels, which subsequently buffers the effects of environmental perturbations on cellular functions (Wu et al., 2009). Thus, some miRNAs evolved to maintain the robust association between gene expression patterns and fixed developmental traits (Peterson et al., 2009).

In contrast to their role on constraining plasticity during development, miRNAs seem to play a role in enhancing plasticity in the context of behavior and neuronal functions. This has been demonstrated in several recent studies, which implicated multiple miRNA genes in the regulation of neuronal plasticity (Fiore et al., 2011; Siegel et al., 2011; McNeill and Van Vactor, 2012). For example, *miR-132* and *miR-134* have been implicated in the growth and pruning of mammalian dendritic spines (Schratt et al., 2006; Impey et al., 2010), *miR-133b* in neurotransmitter vesicle size (Kim et al., 2007), and others in different aspects of neuronal plasticity (Schratt, 2009).

Given the emerging importance of miRNAs for neuronal plasticity, it is perhaps not surprising that distinct miRNA genes have been implicated in the regulation of behavioral plasticity as well, including entrainment of the circadian clock in mammals (Na et al., 2009; Bartok et al., 2013), positive and negative responses to specific odors in *Drosophila* (Li et al., 2013), and the social response to unfamiliar conspecifics in mice (Gascon et al., 2014). Specific miRNA genes have also been implicated in processes associated with learning and memory, including in social insects. For example, expression levels of several miRNAs are associated with spatial learning (Qin et al., 2013), and long-term olfactory memory (Cristino et al., 2014) in the honey bee. In addition, studies in *Drosophila melanogaster* showed that blocking the action of *dme-miR-276a* in the mushroom bodies, a key neuroanatomical structure necessary for many cognitive functions (Heisenberg, 2003), leads to inhibition of long-term olfactory memory formation via direct interaction with the dopamine receptor *DopR* (Li et al., 2013).

In addition to neuronal functions of miRNAs, some miRNAs can also affect behavior via their actions in in non-neuronal tissues. For example, manipulations of the expression of *miR-184* is implicated it in the synthesis and release of insulin (Morita et al., 2013), a conserved and ubiquitously important neuroendocrine factor that is secreted from non-neuronal cells in all animal lineages (Ament et al., 2008; Wolschin et al., 2011).

## The Possible Role of miRNAs in the Regulation of Traits Associated with Eusociality

### Developmental Plasticity: Caste Differentiation

The completion of the honey bee genome revealed many conserved candidate miRNAs (Weinstock et al., 2006). Because of the known functions of miRNAs in the regulation of various developmental processes, it has been suggested that miRNAs are likely to contribute to the developmental processes of reproductive caste (queen-worker) differentiation (Weaver et al., 2007; Bonasio et al., 2010). In this context, it was recently reported that the expression level of the miRNA *ame-miR-71* is higher in workers relative to queens during the pupal stage (Weaver et al., 2007). A subsequent study revealed that many additional miRNAs are differentially expressed between larvae that are destined to develop as either queens or workers (Shi et al., 2014). These differences in miRNA expression levels are consistent with the hypothesis that miRNAs are involved in the regulation of caste determination and differentiation. However, functional analyses of these miRNAs is needed to establish genetic causation between changes in the expression of specific miRNAs and the development of reproductive traits.

In contrast to species such as the honey bee, in which caste differentiation occur early during larval development, in some eusocial species such as the ant *Harpegnathos saltator*, females retain the potential to become reproductive individuals (gamergates) throughout life. Although gamergates are morphologically worker-like, they reproduce and behave like a queen following the loss of the primary queen (Peeters et al., 2000). In this species, the transition of workers into gamergates is associated with a significant reduction in the global expression levels of several miRNA genes (Bonasio et al., 2010). How global miRNA down-regulation occurs, and why it might be important for the regulation of reproductive division of labor in this species, are not yet known.

Surprisingly, recent reports suggest that exogenous miRNAs can also affect reproductive caste-determination in honey bees. Guo et al. (2013) reported that miRNAs are present in the honey bee larval food. A comparison of short RNAs found in worker food versus “royal jelly” (food that induces queen development) indicated that the overall amount of miRNAs that are fed to worker-destined larvae is significantly higher than in food given to queen-destined larvae. Furthermore, queen-destined larvae that were fed with royal jelly supplemented with the worker-enriched miRNA *ame-miR-184* developed some worker-like morphologies (e.g., smaller body and shorter wings). This remarkable finding suggests that in honey bees, the consumption of exogenous miRNAs could play an important role in the differentiation of totipotent larvae into either sterile workers or reproductive queens. In this context, the conserved role of *miR-184* in the regulation of neuroendocrine functions across different animal taxa (Morita et al., 2013) is particularly alluring. In agreement with this hypothesis, genetic pathways that are targeted by *miR-184* in mammals are also important for queen versus worker differentiation in bees (Wolschin et al., 2011; Foret et al., 2012), suggesting that perhaps these observed effects of *miR-184* are conserved to the same pathways across mammals and insects.

### Behavioral Plasticity: Division of Labor

One of the best-studied aspects of eusociality is the division of labor between workers. In some eusocial insects, such as the honey bee, division of labor relates to age (Robinson, 1992; Naug and Gadagkar, 1998; Kim et al., 2012). Young worker bees (typically <14 days of age) typically perform in-hive tasks, such as brood care (“nursing”) or food handling, and later in life (typically at around 3 weeks of age) they transition to foraging outside the hive. This well-characterized form of behavioral development has emerged as an excellent model for the molecular mechanisms involved in social behavioral plasticity (Robinson et al., 1997, 2005; Denison and Raymond-Delpech, 2008; Bloch and Grozinger, 2011). Gene expression studies, mostly using brain tissue, have demonstrated that division of labor in honey bees, and several other eusocial species, is associated with task-specific mRNA transcriptional profiles (Whitfield et al., 2003; Adams et al., 2008; Daugherty et al., 2011; Liu et al., 2011; Oxley et al., 2014).

Three recent studies also examined the possible association between changes in brain miRNAs transcript levels and division of labor in honey bees (Behura and Whitfield, 2010; Greenberg et al., 2012; Liu et al., 2012). All three studies found that the expression levels of several miRNAs are upregulated in the brains of foragers relative to bees that perform in-hive duties (**Table 1**).

**Table 1:** microRNAs that are differentially expressed in honey bee foragers and nurses.

miRNAs	Behura and Whitfield (2010)	Greenberg et al. (2012)	Liu et al. (2012)
*ame-let-7*	Nurses		Nurses
*ame-Bantam*		Foragers	
*ame-miR-9*	Foragers		
*ame-miR-12*	Foragers		
*ame-miR-13a*	Nurses		
*ame-miR-13b*	Nurses		Foragers^*^
*ame-miR-14*	Nurses		
*ame-miR-31a*			Nurses
*ame-miR-92a*	Foragers		Foragers
*ame-miR-124*	Nurses		
*ame-miR-133*			Foragers
*ame-miR-184*		Foragers	
*ame-miR-210*	Foragers		Foragers
*ame-miR-219*	Foragers		
*ame-miR-263*	Foragers		
*ame-miR-275*			Nurses
*ame-miR-276*	Nurses		
*ame-miR-278*			Foragers
*ame-miR-279*			Nurses
*ame-miR-283*	Foragers		
*ame-miR-2796*		Foragers	

miRNAs that were differentially expressed in at least two of the studies are highlighted in red. Denoted worker group (foragers/nurses) expressed significantly higher levels relative to the other group. Behura and Whitfield (2010) measured expression of pri-miRNA using qRT-PCR, Liu et al. (2012) relied on RNA sequencing of mature miRNA, while Greenberg et al. (2012) measured mature miRNA using northern blots. ^*^qRT-PCR analysis showed a trend that was opposite to the RNA-seq data.

The association of miRNA transcript levels with specific behavioral states in colonies of eusocial insects is not limited to reproductive and worker divisions of labor. For example, reproductive queens in diverse eusocial species mate only once in their lifetime (Woyke, 1955). In honey bees, newly eclosed virgin queens (gynes) leave the hive for their sole “nuptial flight” during which they copulate with 10–20 males. After mating, they spend the rest of their lives laying eggs inside the hive. Thus, virgin and mated queens represent two distinct behavioral and physiological states (Winston, 1987). A recent study of the miRNA transcriptome in virgin and mated honey bee queens identified two different genes (*ame-miR-124* and *ame-miR-275*), which are differentially expressed in virgin and mated queens (Wu et al., 2014). While the precise function of these miRNAs in honey bees is not known, previous reports indicate that *miR*-*124* is an evolutionary conserved, brain-enriched miRNA that plays a role in neural development and plasticity in invertebrates, birds, and mammals (Cao et al., 2007; Makeyev et al., 2007; Rajasethupathy et al., 2009), and more specifically in the development and function of the peripheral sensory system in *C. elegans* (Clark et al., 2010). *miR-275* is also conserved across insects, and has been implicated in the regulation of egg laying behavior in *Aedes aegypti* (Bryant et al., 2010). Wu et al. (2014) speculated that the upregulation of *ame-miR-124* miRNA in virgin queens might be related to the modulation of sensory and/or other neuronal functions associated with mating behaviors, while the increased expression of *ame-miR-275* in mated queens might be important for the newly mated queens to initiate egg-laying behavior.

## A Case for the Possible Role of miRNAs in the Evolution of Eusociality

Why eusociality evolved multiple times within Hymenoptera but is rare in other insect orders is still a mystery. Several evolutionary models have attempted to explain this phenomenon by proposing various ultimate selective forces that may have driven the repeated rise of eusocial traits in this insect order (Hamilton, 1964; Andersson, 1984; Nowak et al., 2010). Although the regulation of phenotypes associated with eusociality has been independently linked to key regulatory pathways such as insulin and juvenile hormone signaling (Page and Amdam, 2007; Toth and Robinson, 2007; Bloch and Grozinger, 2011), the actual molecular events that supported traits contributing to eusociality remain elusive. Here, we propose that the molecular evolution of specific miRNAs could have contributed to the phenotypic evolution of eusociality. We propose that these miRNAs may have contributed to the emergence of eusociality by either introducing new regulatory nodes to ancestral behavioral genetic networks, and/or by supporting novel behavioral genetic networks.

The primary sequence of mature miRNAs is often completely conserved across long phylogenetic distances. Consequently, conserved miRNAs are likely to regulate similar target protein-coding genes in distant taxa, and thus support analogous phenotypes across phylogeny (Lee et al., 2007). Given their broad pleiotropic function, novel miRNAs can modify complex developmental or physiological genetic programs. Because of this, it has been suggested by several investigators that, similarly, to the evolution of protein regulatory networks (e.g., evolution of novel transcription factors), novel miRNAs could lead to evolutionary innovations (Sempere et al., 2006; Lee et al., 2007; Niwa and Slack, 2007; Tarver et al., 2012) such as the establishment of new body plans, or novel behavioral traits (Peterson et al., 2009).

**FIGURE 2:**
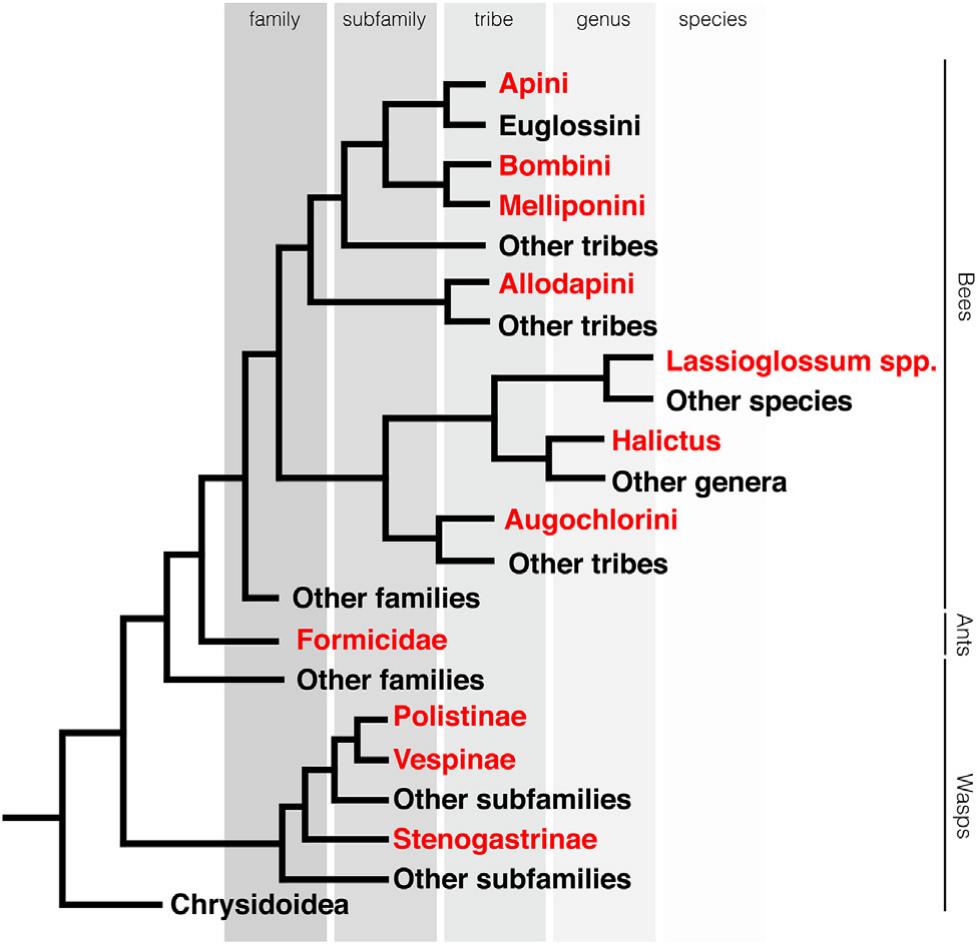
**Eusociality evolved multiple times in hymenoptera**. Phylogeny of the Aculeata. Clades containing eusocial species highlighted in red. Phylogeny is based on Danforth et al. (2013) and Johnson et al. (2013). Each branch represents the lowest taxonomic classification level that is solely comprised of eusocial species.

Consistent with this premise, the evolution of bilateral animals from eumetazoans was associated with a great expansion in the number of miRNA genes (Niwa and Slack, 2007). Other examples include the many novel miRNA genes found within placental mammals, and their clade-specific expansion in primates (Sempere et al., 2006). Although the evolution of eusociality is considered a major evolutionary transition event (Maynard Smith and Szathmary, 1995), the hypothesis that it was also associated with the evolution of novel miRNAs has not been previously suggested. We reasoned that the monophyletic Aculeata clade is ideal for testing this hypothesis since, based on current phylogenetic models (Danforth et al., 2013; Johnson et al., 2013), eusociality has independently emerged in this group multiple times (**Figure 2**). Below we discuss two independent, non-mutually exclusive hypotheses for the possible involvement of miRNAs in the evolution of eusociality.

### Hypothesis 1: Specific miRNAs have been Repeatedly Associated with Eusocial Evolution in Hymenoptera

Here, we hypothesize that, similarly to the evolution of novel transcription factors, the repeated evolution of specific new miRNAs, either *de novo* or via duplication events, facilitated the evolution of some eusocial traits in multiple independent clades that currently display eusociality. Under this hypothesis, novel miRNAs in current eusocial species act as essential nodes in genetic networks that support eusocial traits. If true, we expect that specific miRNAs would be more likely to be present in the genomes of eusocial species in comparison to related solitary species.

**FIGURE 3:**
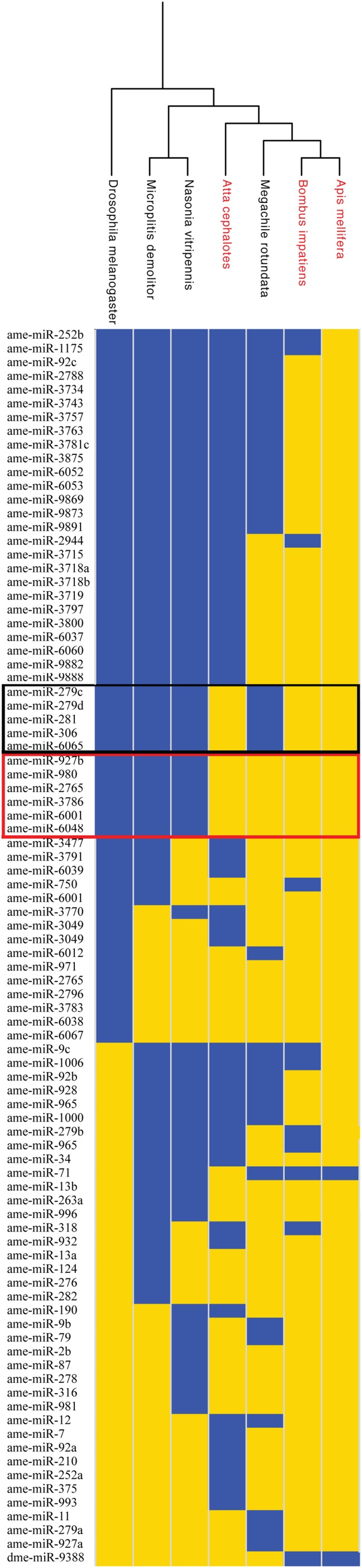
**Phylogenetic distributions of miRNAs in Hymenoptera genomes**. miRNAs present in each genome are shown in yellow, while those absent are shown in blue. miRNAs present in all or only one species are not shown. Data are clustered based on the phylogenetic relationships between the species analyzed, with eusocial species shown in red. Genes framed in black are present only in eusocial species. Genes framed in red are present only in Aculeata. The fruit fly *Drosophila melanogaster* served as the outgroup. Phylogeny based on Danforth et al. (2013) and Johnson et al. (2013).

As an initial test of this hypothesis, we searched for miRNA genes in the sequenced genomes of species in the Aculeata clade, which includes all living eusocial species in Hymenoptera. We first generated a list of all known annotated miRNA genes available in miRBase for the eusocial honey bee *Apis mellifera*, the solitary wasp *Nasonia vitripennis*, and the fruit fly *D. melanogaster* (Griffiths-Jones et al., 2006). Next, we searched for the presence or absence of each annotated miRNA in several representative hymenopteran genomes (**Table 2**) using BLASTN (Altschul et al., 1997). We only scored a miRNA as “present” if an exact match to the mature miRNA sequence was found in the genome (**Figure 3**). Consistent with data from other animal clades (Tarver et al., 2012), we found that most annotated miRNA genes aligned with phylogeny rather than with the presence or absence of eusociality. Nevertheless, five miRNA genes (*ame-miR-281*, *ame-miR-306*, *ame-miR-279c*, *ame-miR-279d*, and *ame-miR-6065*) seem to be associated with the expression of eusocial traits independent of phylogeny (**Figure 3**).

**Table 2:** Genomes analyzed.

Order	Species	Common name	Eusocial	NCBI BioProject ID
Ixodida	*Ixodes scapularis*	Deer tick	No	34667
Hemiptera	*Acyrthosiphon pisum*	Pea aphid	No	29489
Coleoptera	*Tribolium castaneum*	Red flour beetle	No	15718
Lepidoptera	*Bombyx mori*	Silkworm	No	205630
Diptera	Drosophila melanogaster^*^	Fruit fly	No	164
Diptera	*Aedes aegypti*	Mosquito	No	19731
Hymenoptera	*Athaliae rosae*	Turnip sawfly	No	167403
Hymenoptera	Microplitis demolitor^*^	Parasitoid wasp	No	251518
Hymenoptera	Nasonia vitripenis^*^	Parasitoid wasp	No	20073
Hymenoptera	*Nasonia longicornis*	Parasitoid wasp	No	20225
Hymenoptera	*Nasonia girulta*	Parasitoid wasp	No	20223
Hymenoptera	Apis meliffera^*^	Honey bee	Yes	13343
Hymenoptera	***Apis dorsata***	Honey bee	Yes	174631
Hymenoptera	***Apis florea***	Honey bee	Yes	86991
Hymenoptera	Bombus impatiens^*^	Bumble bee	Yes	70395
Hymenoptera	***Bombus terrestris***	Bumble bee	Yes	68545
Hymenoptera	***Lasioglossum albipes***	Sweat bee	Facultative	174755
Hymenoptera	Megachile rotundata^*^	Leafcutter bee	No	87021
Hymenoptera	***Harpegnathos saltator***	Jumping ant	Yes	50203
Hymenoptera	***Camponotus floridanus***	Carpenter ant	Yes	50201
Hymenoptera	Atta cephalotes^*^	Leafcutter ant	Yes	48091
Hymenoptera	***Solenopsis invicta***	Fire ant	Yes	49629
Hymenoptera	***Pogonomyrmex barbatus***	Harvester ant	Yes	45797
Hymenoptera	***Polistes dominula***	Paper wasp	Yes	Unpublished

The following genomes were analyzed for the presence or absence of miRNAs. We performed an initial BLAST search of annotated miRNAs from D. melanogaster, A. mellifera, and N. Vitripenis in the species denoted by ^*^. Candidate miRNAs identified as either present only in the genomes of eusocial species (red) or only in Aculeate species (bold), were subsequently analyzed in all genomes listed.

**FIGURE 4:**
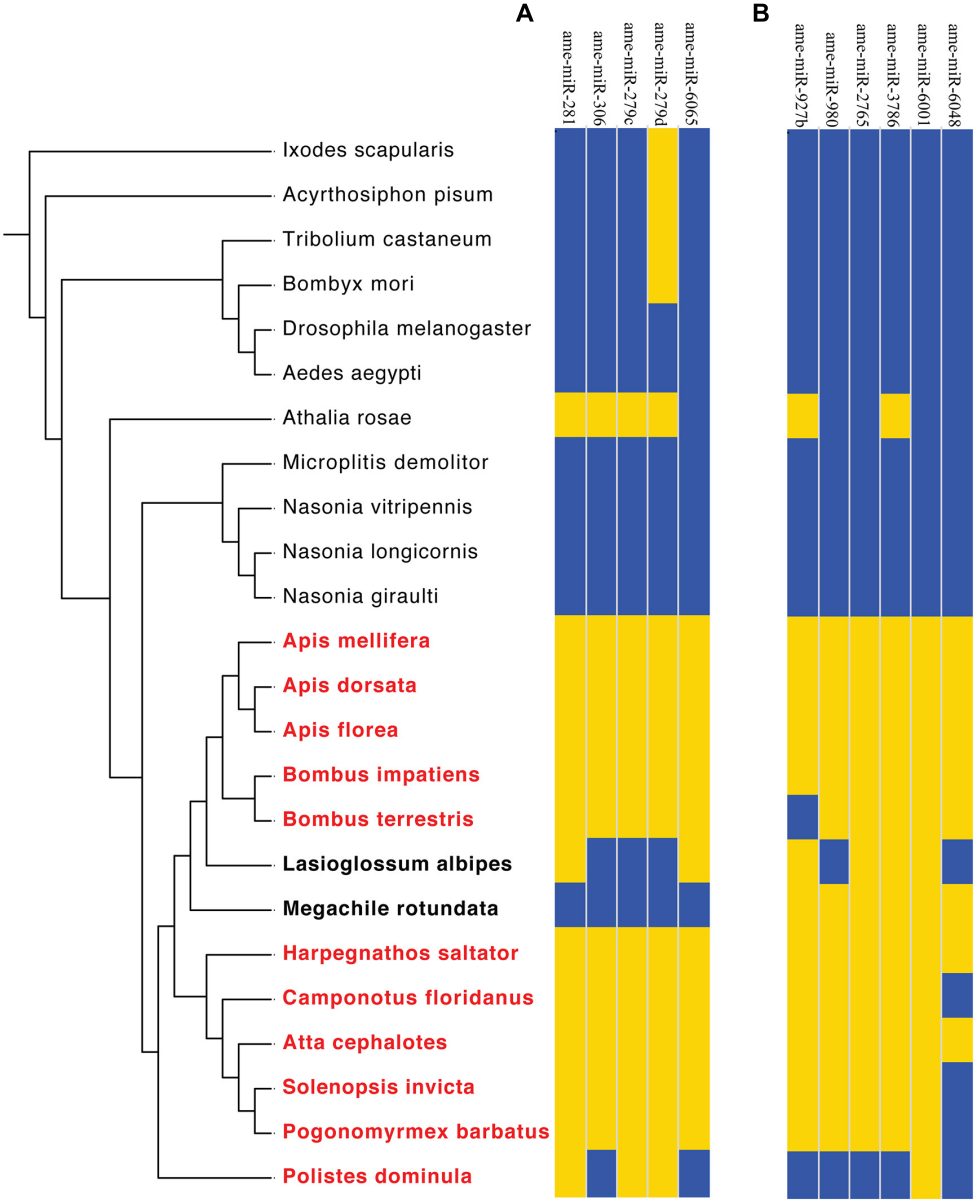
**Phylogenetic distributions of Aculeata-specific miRNA**. miRNAs present in each genome are shown in yellow, while those absent are shown in blue. **(A)** High resolution analysis of eusociality-associated candidate miRNAs from **Figure 3**. **(B)** High resolution analysis of Aculeata-spcific candidate miRNAs. Data are clustered based on the phylogenetic relationships between the analyzed genomes, with Aculeate species in bold and eusocial species in red. *L. albipes* is facultative eusocial. Species phylogeny as in **Figure 3**.

To further refine our results, we subsequently extended the bioinformatic analyses to all available sequenced hymenopteran genomes, as well as several non-hymenopteran insect species, which served as outgroups (**Figure 4A**). Although the low sequence coverage for some of the analyzed ant genomes could lead to higher false-negative discovery rate, we reasoned that the likelihood that certain miRNAs will be falsely missing from all analyzed genomes is very low. Future miRNA sequencing data from many of the species studied here should further help reducing the possibility of false-negatives.

This analysis revealed that three out of the five putative eusociality-associated miRNAs were unique to Hymenoptera (*ame-miR-281, ame-miR-306,* and *ame-miR-279c*), and one possibly unique to Aculeata (*ame-miR-6065*). The phylogenetic distribution of these five miRNAs indicated that multiple eusociality-associated miRNAs might have been gained and lost during the Hymenoptera radiation. In addition, we found that two eusociality-associated miRNA genes (*miR-306* and *miR-6065*) were lost in the eusocial wasp *Polistes dominula*. Markedly, two of the eusocial-related miRNAs (*miR-281* and *miR-6065*) were also present in the genome of the facultative eusocial bee *Lasioglossum albipes*. One possible explanation for this finding is that these specific miRNAs are important for traits associated with basal levels of sociality such as communal living, overlapping generations, and reproductive division of labor (Kocher et al., 2013).

Our analysis also revealed that two of the candidate eusociality-related miRNAs (*mir-279c* and *mir-279d*) belong to a single conserved miRNA-family (Cayirlioglu et al., 2008; Hartl et al., 2011; Luo and Sehgal, 2012; Mohammed et al., 2014). The most parsimonious interpretation of these observed phylogenetic patterns is that *miR-279d* is conserved across Arthropoda, but was lost in Diptera and Hymenoptera, and then reappeared via duplications in eusocial Aculeates. In contrast, *miR-279c* seems to have specifically evolved in Hymenoptera prior to the divergence of Aculeata, and was subsequently lost from non-social Aculeate species. The identification of members of the *mir-279* family as possible candidate genes for the evolution of eusociality is in agreement with findings about their differential regulation between nurses and foragers (**Table 1**), and possible functions in *Drosophila*. For example, members of the *mir-279* family have been implicated in regulating neuronal development (Hartl et al., 2011), olfactory receptivity (Cayirlioglu et al., 2008; Hartl et al., 2011), and circadian rhythms (Luo and Sehgal, 2012). It is interesting to note that plasticity in both circadian rhythms (Bloch, 2010) and olfactory neurons has been shown to be associated with worker and reproductive divisions of labor in eusocial Hymenoptera (López-Riquelme et al., 2006; Zube and Rössler, 2008; Mysore et al., 2009). Although preliminary, these findings suggest that members of the *mir-279* gene family are prime candidates for studies on the possible roles of specific miRNA in the evolution of eusociality-related traits.

To further test this hypothesis it will be necessary to increase the phylogenetic resolution of our analyses by studying the miRNA repertoire encoded by the genomes of additional social and solitary insects. It will also require the development of tools that will allow the manipulation of focal miRNA expression to causally determine their effect on behavioral and physiological traits related to eusociality. The recent progress in genome-editing techniques for honey bees and other social insects (Wang et al., 2013; Schulte et al., 2014) suggest that this will be feasible in the near future. Another complementary approach will be to study the protein-coding genetic networks that eusociality-associated miRNAs are interacting with. By identifying the genes involved, their spatial and temporal expression patterns, and the possible physiological and behavioral processes they modulate, a higher resolution picture of the genetics that support eusociality could emerge.

### Hypothesis 2: Aculeate-Specific miRNAs were Required for Eusocial Evolution

The second hypothesis we consider is that the presence of specific miRNAs in the pre-eusocial Aculeate genome might have “primed” certain species to evolve eusociality. In other words, specific miRNAs, already present in the genome of the solitary Aculeate ancestor were required, but not sufficient, for the emergence of eusocial traits. Under this hypothesis, specific miRNAs already present in the ancestral solitary aculeate increased the probability of emergence of specific behavioral and physiological traits in response to selective pressures that favored eusociality.

If true, we expect that specific miRNAs should be present in all Aculeate genomes, but absent from all other hymenopteran genomes, as eusociality has never been observed in hymenopteran species outside of the Aculeta. Our initial analysis revealed six Hymenoptera specific miRNA genes (*ame-miR-927b*, *ame-miR-980*, *ame-miR-2765*, *ame-miR-3786*, *ame-miR-6001*, and *ame-miR-6048*; **Figure 3**). However, two of these genes were were also present in the sawfly *Athalia rosae* (*ame-miR-927b* and *ame-miR-3786*), and therefore are not specific to Aculeata. Three additional genes (*ame-miR-980*, *ame-miR-2765*, and *ame-miR-6048*) appear to have originated after the divergence of Vespidae and therefore did not fulfill the above criteria (**Figure 4B**). Thus, our analysis revealed *ame-miR-*6001 as the single Aculeate-specific miRNA candidate gene that should be tested in the context of the above hypothesis. Similarly to Hypothesis 1 (see Hypothesis 1: Specific miRNAs have been Repeatedly Associated with Eusocial Evolution in Hymenoptera), the possible role of *miR-6001* in the repeated evolution of eusocial traits in Aculeata is hypothetical. Directly testing the hypothesis we put forward here will require extensive molecular, biochemical, and phenotypic studies of its possible physiological and behavioral roles in eusocial traits.

## A Look to the Future

To date, the majority of data about the function of miRNAs in social insects come from studies of the European honey bee, *A. mellifera*. Thus, additional molecular and evolutionary analyses of non-hymenopteran eusocial insects as well as other eusocial and solitary clades in the Hymenoptera are required in order to better understand miRNA functions in the context of eusociality. Furthermore, to establish causation between the action of specific miRNAs and eusocial traits, new *in vivo* genetic and molecular techniques to manipulate social insects are required. Recent advances in molecular genetics of social insects (Yan et al., 2014), and the successful generation of transgenic honey bees (Ben-Shahar, 2014; Schulte et al., 2014), suggest that such studies might be possible in the near future. Furthermore, the development of pharmacological reagents that can block or mimic the action of specific miRNAs (e.g., antagomirs), would represent another important step in that direction (Cristino et al., 2014).

## Author Contributions

ES collected and analyzed genomic data. ES, GB, and YB-S gathered and synthesized relevant literature. ES, GB, and YB-S wrote the manuscript.

## Conflict of Interest Statement

The authors declare that the research was conducted in the absence of any commercial or financial relationships that could be construed as a potential conflict of interest.
